# Assessing the Relative Stability of Dimer Interfaces in G Protein-Coupled Receptors

**DOI:** 10.1371/journal.pcbi.1002649

**Published:** 2012-08-16

**Authors:** Jennifer M. Johnston, Hao Wang, Davide Provasi, Marta Filizola

**Affiliations:** Department of Structural and Chemical Biology, Mount Sinai School of Medicine, New York, New York, United States of America; University of Illinois, United States of America

## Abstract

Considerable evidence has accumulated in recent years suggesting that G protein-coupled receptors (GPCRs) associate in the plasma membrane to form homo- and/or heteromers. Nevertheless, the stoichiometry, fraction and lifetime of such receptor complexes in living cells remain topics of intense debate. Motivated by experimental data suggesting differing stabilities for homomers of the cognate human β1- and β2-adrenergic receptors, we have carried out approximately 160 microseconds of biased molecular dynamics simulations to calculate the dimerization free energy of crystal structure-based models of these receptors, interacting at two interfaces that have often been implicated in GPCR association under physiological conditions. Specifically, results are presented for simulations of coarse-grained (MARTINI-based) and atomistic representations of each receptor, in homodimeric configurations with either transmembrane helices TM1/H8 or TM4/3 at the interface, in an explicit lipid bilayer. Our results support a definite contribution to the relative stability of GPCR dimers from both interface sequence and configuration. We conclude that β1- and β2-adrenergic receptor homodimers with TM1/H8 at the interface are more stable than those involving TM4/3, and that this might be reconciled with experimental studies by considering a model of oligomerization in which more stable TM1 homodimers diffuse through the membrane, transiently interacting with other protomers at interfaces involving other TM helices.

## Author Summary

G Protein-Coupled Receptors (GPCRs) are the largest family of membrane proteins targeted by drugs in clinical practice. Despite being at the forefront of biomedical research for many years, there is still considerable uncertainty about how GPCRs function at a molecular level. Although substantial evidence exists in support of their association in cell membranes, it is unclear how general and/or long-lasting this phenomenon is and whether it plays a significant role in GPCR function. This observation highlights the importance of understanding the rules that govern receptor-receptor interactions in living cells. Here, we report the results of computer simulations from which we estimated the relative stability of dimers formed by different, yet highly homologous, prototypic GPCRs. Our results suggest overall transiency in receptor-receptor interactions at the simulated different dimerization interfaces, but a variable strength of association depending on the specific residue composition or shape of the interface. The methodology we propose is expected to provide a level of molecular detail that is unattainable using current experimental techniques. Our ultimate goal is to generate unique hypotheses of receptor-receptor inter-helical interactions that can be tested experimentally to help elucidate the role of receptor association in GPCR function.

## Introduction

G Protein-Coupled Receptors (GPCRs) have been reported to associate in the cell membrane to form dimers/oligomers. While incontrovertible evidence exists for the constitutive dimerization of disulfide-linked family C GPCRs [1], the interpretation of oligomerization studies of members of the largest subfamily A of GPCRs [2] has often been difficult and controversial [3], [4], [5], [6], since the majority of the techniques used to infer GPCR association in living cells are unable to conclude unambiguously in favor of direct physical interaction between receptors. Most importantly, very few GPCR oligomerization studies have been able to provide any information about the fraction of receptors that are interacting at a given time or the corresponding dynamics of the interactions, rendering it impossible to determine, with any certainty, which molecular species (i.e. individual protomers, dimers, or higher-order oligomers) signal through interaction with intracellular proteins. These uncertainties have fueled an ongoing debate regarding the physiological role of GPCR oligomerization, exacerbated by the evidence that individual GPCR protomers, when reconstituted into nanodiscs, can signal to G proteins [6], [7], [8], [9].

Recent studies using single-molecule approaches have begun to address the details of the spatial and temporal organization of GPCR complexes in living cells. Single-molecule total internal reflection fluorescence microscopy (TIR-FM) was recently used to track the position of individual molecules of the M1 muscarinic acetylcholine receptor (M1R) labeled with fluorescent M1R antagonists in living cells [10]. Both single- and dual-color imaging experiments suggested a transient (∼0.5 seconds) formation of M1R dimers and a dimeric fraction of only ∼30% dimers at any given time. Although similar conclusions were reached by a single-molecule study of another family A GPCR, i.e the N-formyl peptide receptor [11], the possibility cannot be excluded that the fluorescent ligands used to image the single molecules in both studies might have altered the lifetime and preferred stoichiometry of the observed GPCR oligomers. It remains to be determined whether or not the features highlighted in these studies are the same for all GPCRs, or just specific subtypes.

Recent fluorescence recovery after photobleaching (FRAP) studies of human β1 and β2-adrenergic receptors (B1AR and B2AR, respectively) [12] have raised the possibility that the strength of GPCR association may vary significantly, even among closely related receptor subtypes. Although the antibody-mediated capping approach used to immobilize receptors in these studies may have affected the interpretation of the results, B1AR was suggested to interact transiently (on a timescale of seconds) whereas B2AR appeared to form more stable complexes (on a timescale of minutes).

Fung and colleagues [13] reported data in support of spontaneous B2AR oligomerization using Förster resonance energy transfer (FRET) between relatively small fluorescent probes attached to purified B2AR reconstituted into phospholipid vesicles. The authors hypothesized predominant tetrameric arrangement for the B2AR, although they did note the difficulty of unambiguously determining the stoichiometry of receptor oligomers from a reconstituted system. Additional FRET saturation studies showed greatest energy transfers for H8 and smallest for TM6, based on which, the authors proposed a preferential oligomeric arrangement of B2AR involving TM1 and H8 at the interface, similar to that previously suggested for the dopamine D2 receptor from a combination of molecular modeling and cysteine cross-linking experiments [14]. Further support for the simultaneous involvement of helices TM1 and H8 at an interface was recently provided by chemical cross-linking of endogenous cysteines in rhodopsin in disk membranes [15]. Several additional experimental studies support the direct primary involvement of TM1, as well as TM4 in GPCR oligomerization under physiological conditions [16]. Although an alternative dimerization interface involving both helices TM5 and TM6 was recently suggested by the crystal structures of the chemokine CXCR4 [17] and μ-opioid [18] receptors, its physiological relevance has not yet been demonstrated. Here, we sought insight into the dimerization free energy of models of human B1AR and B2AR based on high-resolution inactive crystal structures interacting at two putative interfaces involving TM1/H8 or TM4/3, using biased molecular dynamics (MD) simulations. Specifically, we combined umbrella sampling and metadynamics simulations to provide hypotheses of the role played by the interface sequence and configuration in imparting stability to the specific dimeric arrangements that we have simulated for both B1AR and B2AR. These studies provide a mechanistic insight into the association of GPCRs at putative dimerization interfaces at a level of molecular detail that is unattainable using current experimental techniques, yet crucial to guiding future experiments aimed at exploring the role of dimerization in receptor function.

## Results

**Figure 1:**
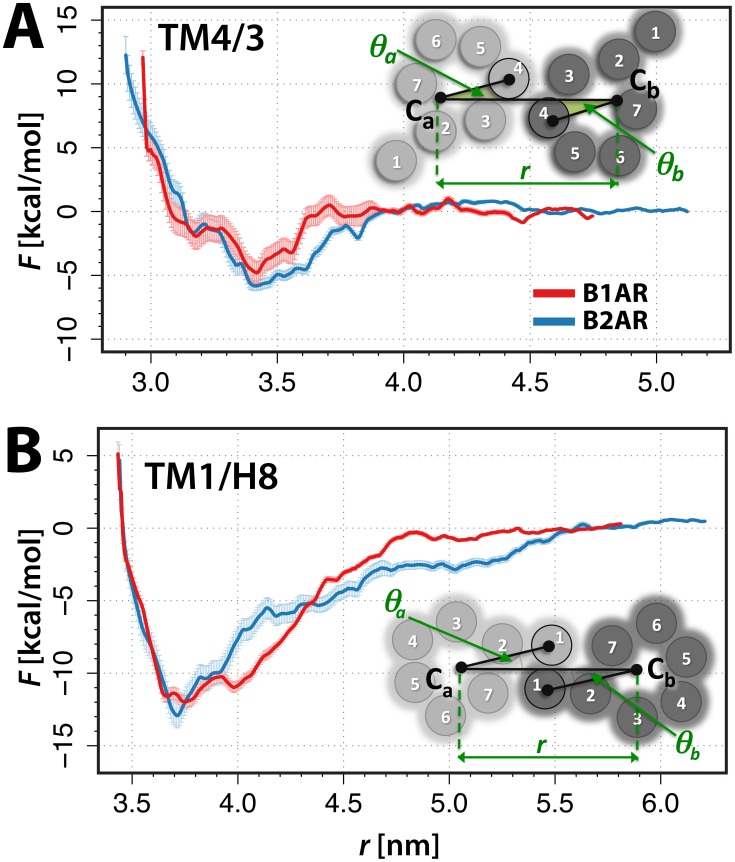
Free energy surface (FES) as a function of protomer separation. Panel (A) shows results obtained for the TM4/3 interface of B1AR (red) and B2AR (blue) homodimers. The monomeric state was defined as being in the *r* = 4.5–4.8 nm range, and was assigned a free energy of zero. Panel (B) shows the FES as a function of the distance between the centers of mass of the protomers at the TM1 interface formed by (red) B1AR and (blue) B2AR, with the monomeric state assigned as *r* = 5.5–5.9 nm. The error bars are given for each case, and are calculated as described in Text S1. In each panel, the inset shows a schematic representation of the CVs (*r*, *θ_a_* and *θ_b_*) in each of the interface arrangements.

The results presented herein derive from approximately 160 microseconds (µs) of coarse-grained (CG), biased MD simulations. Two different dimeric arrangements were considered for the B1AR and B2AR. Specifically, these correspond to the interfaces illustrated schematically in the insets of Fig. 1, and are named TM4/3 and TM1/H8 after the helical domains involved in symmetric interactions. Notably, there can be different types of TM1/H8 interfaces depending on whether the TM1 residues involved in symmetric interactions are adjacent to TM7 or TM2. We focused on the interface with TM1 residues adjacent to TM7 based on inferences from recent cross-linking experiments of a family A GPCR [14]. We also note that this particular packing of TM1, simultaneously involving H8, is not possible using a rhodopsin structural template [14]. The corresponding MARTINI-based CG representations of these dimeric configurations were embedded in an explicit, CG, hydrated 1-palmitoyl-2-oleoyl-sn-glycero-3-phosphocholine (POPC)/10% cholesterol model membrane, resulting in approximately 30,000 particles. Following earlier studies [19], [20], we used three collective variables (CVs) to describe the dimeric arrangements recapitulated in the inset of Fig. 1A. Briefly, these CVs describe the separation (*r*) between the centers of mass (COMs) of two interacting protomers *a* and *b*, i.e., C*_a_* and C*_b_*, and their relative orientation, described by the relative rotational angles *θ_a_* and *θ_b_*.

Thorough exploration of the TM4/3 and TM1/H8 interfaces of B1AR and B2AR homodimers was achieved through a pair of biasing forces applied to the CVs, i.e. a harmonic umbrella restraint applied to the separation (*r*) between the COMs of each of the protomers, and a history-dependent Gaussian bias applied to a pre-defined rotational angle range. POPC and cholesterol exchange at the interfaces was evaluated following the procedure described in Text S1 (see also Figs. S1 and S2) to exclude the possibility of desolvation problems at the protomer-protomer interface. Unbiased free energies were subsequently obtained as a function of the separation of the protomers, and of the angle exploration, as described in *Materials and Methods*. A theoretical framework, identical to that previously described by us in [19], was applied to derive the relative dimerization free energies and dimer lifetimes for each system, at the different interfaces. Results are presented in Table 1. A representative CG structure for each system was extracted from the energetic minimum in both *r* and (*θ_a_, θ_b_*) space and converted to an atomistic representation. Each structure was subsequently simulated for 1 ns in an explicitly represented, solvated POPC/10% cholesterol bilayer, and the details of the contacting residues at the interface were derived.

**Table 1:** Results of the free energy calculations of adrenergic receptor dimers.

System	Interface	F(*r*_min_) (kcal/mol)	K_D_ (µm^2^)	ΔG_X_^o^ (kcal/mol)	Lifetime (s)
B1AR	TM4/3	−4.8 (−5.6,−4.0)	3.1 10^−5^ (7.3 10^−6^, 1.4 10^−4^)	−2.3 (−1.5, −3.2)	1.3 10^−4^ (3.2 10^−5^, 5.9 10^−4^)
B2AR	TM4/3	−5.8 (−6.2,−5.5)	3.8 10^−4^ (3.3 10^−4^, 5.0 10^−4^)	−3.7 (−3.3, −4.1)	1.4 10^−3^ (6.9 10^−4^, 2.7 10^−3^)
B1AR	TM1/H8	−12.0 (−12.4,−11.6)	7.2 (3.6,14.3)	−9.7 (−9.2,−10.0)	31.4 (5.8,62.7)
B2AR	TM1/H8	−12.9 (−13.7,−12.1)	13.8 (3.2,60.0)	−10.0 (−9.2, −10.9)	60.5 (13.9,263.1)

Listed are the depths of the minima in the FES, along with the calculated dimerization constant (K_D_), the standard free energy change ΔG_X_°, and the estimated lifetime of each simulated B1AR and B2AR dimer. Confidence intervals are given in parentheses.

### Receptor Protomers Interacting at TM4/3

Fig. 1A shows the reconstructed free energy surface (FES) as a function of the separation, *r*, between the COMs of the protomers for the B1AR (red) and B2AR (blue) homodimers. We note that the overall shapes of the corresponding curves are similar and their depths are equivalent, within the calculated error bars. Upon offsetting the curves to a zero value in the region beyond which the protomers were seen not to be interacting and were therefore designated as monomeric states (*r* = 4.5–4.8 nm), we observe that the depths of each of the two minima are similar between the B1AR and B2AR homodimers. To confirm the choice of the reference state, we sampled one of the systems, specifically the B2AR interacting at the TM4/3 interface, to larger separation distance (Fig. 1). As reported in Table 1, the primary minimum is at −4.8 kcal/mol and −5.8 kcal/mol for B1AR and B2AR, respectively. Using the same theory described in [19], and the equations 1–[Disp-formula pcbi.1002649.e002] 3 reported in *Materials and Methods*, dimerization free energies (i.e., mole-faction standard state free energy changes ΔG_X_°) of −2.3 kcal/mol and −3.7 kcal/mol were calculated for B1AR and B2AR, respectively.

**Figure 2:**
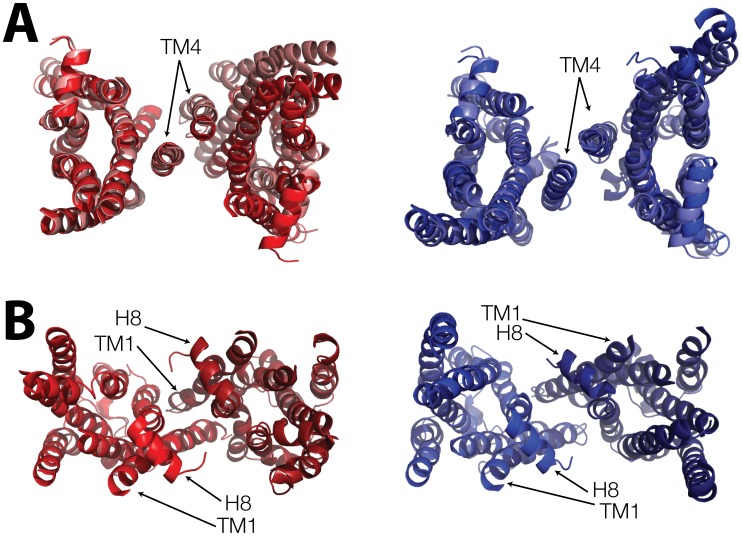
Atomistic structural representations of representative minima of B1AR and B2AR homodimers for the different interfaces. Panel A shows a view from the cytoplasmic side of the minima Θ1 (dark shades) and Θ2 (lighter shades) for the B1AR (red/pink) and B2AR (blue/light blue) at the TM4/3 interface. The receptors are represented as helices with the loops removed for clarity, and are aligned on the left-most protomer. This panel highlights the small difference between the structures at the same separation but with slightly different angle minima. Panel B shows a view from the cytoplasmic side, of the minima extracted from the FES for the TM1/H8 interface. B1AR is shown in red and B2AR is shown in blue. The intracellular loops are omitted for clarity, and H8 and TM1 are highlighted to indicate the packing of the helices at the interface. Contacting residues are listed in Table S1 and depicted in Fig. S4.

We proceeded to identify the relevant orientations of the protomers within each of these dimeric minima by comparing the FES calculated as a function of the angles (see Fig. S3) at *r* = 3.42–3.48 nm for B1AR and *r* = 3.42–3.46 nm for B2AR. The FES as a function of the angle for the TM4/3 interface indicates minima situated approximately at Θ1(*θ_a_,θ_b_*) = (0.2,0.4) (or the symmetric Θ1'(*θ_a_, θ_b_*) = (0.4,0.2) value) and Θ2(*θ_a_,θ_b_*) = (0.45,0.45) radians, respectively. Superpositions of energetically-optimized, all-atom reconstructions of representative structures of the Θ1 and Θ2 minima, obtained using the procedure described in the *Materials and Methods* section, are shown in Fig. 2A for both B1AR (red/pink respectively) and B2AR (blue/light blue). Symmetric inter-helical contacts, defined as average interaction distances between residue Cβ atoms less than or equal to the threshold distance of 11 Å during 1 ns unbiased all-atom MD simulations are listed for each of these representative structures in Table S1. Fig. S4A shows the location of the corresponding residues involved in these contacts.

### Receptor Protomers Interacting at TM1/H8

Fig. 1B shows the FES resulting from the calculation of B1AR and B2AR at interfaces involving TM1/H8 as a function of their separation, *r*, and calculated using the CVs illustrated in the inset of Fig. 1B. As for the TM4/3 interface, the overall shape and depth of the corresponding curves are similar, albeit not identical, between the B1AR and B2AR dimers, most likely due to slight structural divergences between the corresponding reference structures. For the TM1/H8 interface, the monomeric state was defined to be *r* = 5.5–5.9 nm. The primary minimum for each system at this interface indicates a separation between the protomers of ∼3.7 nm, corresponding to −12.0 kcal/mol and −12.9 kcal/mol for B1AR and B2AR, respectively. The ΔG_X_° of the TM1/H8 dimers is approximately −9.7 kcal/mol for B1AR and −10.0 kcal/mol for B2AR. The reconstructed atomistic TM1/H8 dimers of B1AR and B2AR obtained in the same way as previously described, for the TM4/3 interface, are shown in Fig. 2B. Once again, the relative orientations of the protomers in these specific configurations involving the TM1/H8 interfaces were determined from the FES shown in Fig. S3 as a function of the angles, *θ_a_* and *θ_b_*, at *r* = 3.72–3.77 nm for B1AR and *r* = 3.68–3.72 nm for B2AR. The minima are approximately situated at Θ1(*θ_a_,θ_b_*) = (0.45–0.50, 0.45–0.50). Contacting residues between the protomers during 1 ns of explicit simulation are listed in Table S1 and depicted in Fig. S4B.

## Discussion

The spatial and temporal organization of GPCRs in living cells is currently the subject of lively discussion. Although recent applications of single-molecule approaches are beginning to address the preferred stoichiometry, lifetime, and ratio of GPCR dimeric/oligomeric complexes to individual protomers in living cells, they are unable to provide the molecular details of receptor association. Biased MD simulations of the type reported here ensure thorough exploration of the interface for GPCR complex systems in an explicit lipid bilayer, that can be used to draw conclusions about relative values of dimerization free energies and dimer lifetimes.

We have carried out free energy simulations of different GPCR subtypes interacting at two different interfaces that have been suggested, by experiment, to form dimers under physiological conditions. The goal of this study was not to predict the most stable interfaces of dimerization for the GPCR systems studied, which would have required comparison of all possible interfaces, but rather to investigate the effect of different sequences and/or interface configurations on the strength of GPCR dimerization at two dimeric interfaces inferred to be relevant under physiological conditions.

Our study indicates that interfaces involving TM1/H8 are the most stable and the most long-lived (minutes) of the two simulated interfaces for B1AR and B2AR homodimers, based on estimates derived from the calculated free energies. The orientation of the protomers in the TM1/H8 interfacial arrangement is consistent with the close interaction of R333 in H8 (at 13 Å in our dimeric model), which is the residue at which the greatest energy transfer was observed in the recent FRET study of B2AR [13] suggesting spontaneous B2AR oligomerization. The dimer corresponding to the TM4/3 interface appears to be significantly more transient (hundreds of microseconds to milliseconds) than the TM1/H8 interface for both receptors.

The similar lifetimes estimated for B1AR and B2AR homodimers interacting at each of the interfaces tested suggest little difference in temporal organization between these two receptors, sharply contrasting with implications of the recent FRAP studies of human B1AR and B2AR [12] that motivated the present work. However, the possibility cannot be ruled out that the antibody-mediated capping approach used to immobilize receptors in the FRAP study might have caused the B2AR and B1AR to prefer interaction at different interfaces. While the estimated longer lifetime (minutes) of the B2AR dimer involving TM1/H8 at the interface may be considered in line with the views of the aforementioned FRAP study [12], the observation contrasts with inferences from other FRAP studies on dopamine D2 receptors [21], as well as conclusions of recent single molecule studies on muscarinic [10] or N-formyl peptide receptor [11] dimers, which suggest more transient interactions between GPCRs. Although we cannot set apart the contribution of the different membrane environments, we suggest that the results of our simulations may be reconciled with those experimental observations that imply only short-lived interactions by proposing a model of diffusion that features more stable receptor dimers, with TM1 at the interface, diffusing through the membrane and interacting transiently with one another at interfaces involving other TMs, to form short-lived tetrameric or higher-order arrangements.

**Figure 3:**
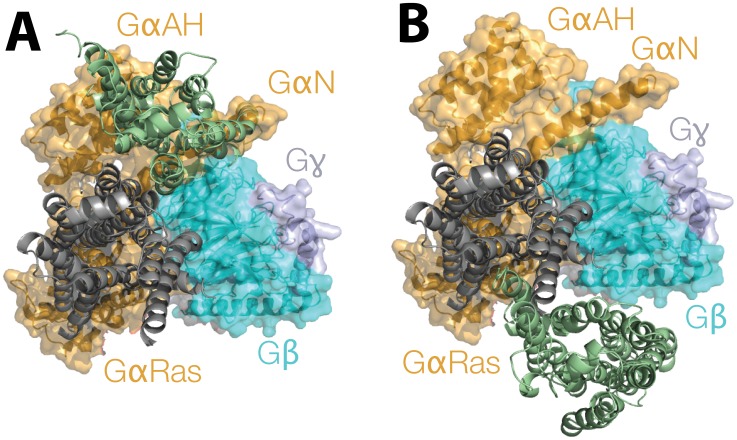
Hypothetical arrangements of B2AR dimers interacting with the Gs protein heterotrimer. The extracellular view of the B2AR-Gs protein complex (PDB ID: 3SN6) is shown with B2AR in grey cartoon representation, and the Gs heterotrimer, is shown in both cartoon and transparent surface (α is in orange, β is in cyan and γ is in pale blue). The first protomer of each of our minimum energy dimers for B2AR (Θ2 for TM4/3) is superimposed on the B2AR from the crystal structure 3SN6, and the position of the second protomer is shown in a green cartoon representation. An interface involving TM4 would favor an exclusive interaction of the dimer with the GαsAH subunit of the G protein, very close to the Gβ subunit when the interface is TM4/3 (panel A). In contrast, with TM1/H8 at the interface, the second protomer would not be involved in significant interactions with any of the G-protein subunits (in B).

By superimposing the TM region of one of the two protomers of the simulated B2AR dimers on the active B2AR TM region of the recent crystal structure of the B2AR- Gs complex (PDB ID: 3SN6 [22]) we note interactions of the second protomer with the G-protein vary, depending on the specific dimeric arrangement of B2AR (Fig. 3). An interface involving TM4/3 would favor an exclusive interaction of the B2AR dimer with the alpha-helical domain of the nucleotide binding Gα subunit of the Gs protein (“GαAH” in Fig. 3A). In contrast, in a B2AR dimeric arrangement with TM1/H8 at the interface, the second protomer would not be involved in significant interactions with any of the G-protein subunits (Fig. 3B). It must be noted that these proposed conformations of the B2AR dimer in complex with the Gs were derived from simple superimposition, and thus would require additional simulations, beyond the scope of this study, to relieve the steric clashes arising from our use of the inactive conformation of the B2AR within the dimeric configurations.

In summary, we have developed a protocol to assess the relative stability of GPCR dimers comprised of protomers interacting symmetrically at different TM regions that is robust within the standard caveats that apply to using a MARTINI-based CG model of proteins and membranes. We have recently published evidence for both small membrane dimeric systems [23] and GPCRs [20] that our CG simulations produce estimates of relative dimerization free energies that are in line with experimental data. Additional validations of the MARTINI model have been independently reported in the literature (e.g., see [24], [25], [26]). We herein demonstrate the dependence on the relative orientation of the protomers, in as much as the FES as a function of protomer separation is significantly different for B1AR and B2AR at interfaces involving either TM4 or TM1. Although the free energy and lifetime estimates reported herein are somewhat dependent on the nature of the starting crystal structures, our calculations appear to be consistent for the different systems we have reported, and within the limits of the theories we have employed. While this manuscript was under review, a paper reporting further elaboration of simulation protocols used to study the self-assembly of rhodopsin molecules [27], and now coupled with umbrella sampling calculations similar to those we published on delta opioid receptor [19], [20], has appeared in the literature [28]. Notably, the conclusions of these independent simulations about the relative stability of dimerization interfaces are in agreement with our calculations.

We are confident that the protocol described herein can be generalized to begin deciphering the mechanistic details of dimerization of other GPCRs at a level of molecular detail that is unattainable using current experimental techniques. Although we do not expect to obtain an exact correspondence between the estimated lifetimes of GPCR dimers and those measured experimentally, our assessment of relative strength of association at different interfaces can be used constructively to predict specific interactions at the dimerization interface that might aid the design of experiments to assess the role of dimerization in receptor function.

## Materials and Methods

### Initial Model Systems

Initial molecular models of human B1AR and B2AR were built using available inactive crystal structures of the turkey B1AR and human B2AR (PDB identification codes: 2VT4 [29], chain B, and 2RH1 [30], respectively) as structural templates. First, missing segments in the B1AR and B2AR crystal structures were built using Rosetta [31]. Specifically, these segments corresponded to sequence fragments 256–260, 306–310, and 313–317 in the B1AR and the intracellular loop 3, sequence fragment 231–262, which had been replaced by a T4 lysozyme in the B2AR crystal structure. To restore the broken ionic lock between TM3 and TM6 in the B2AR crystal structure, a standard MD simulation of 100 ns was carried out after embedding the receptor into an explicit hydrated POPC/10% cholesterol bilayer, following the procedure described in [32]. The homology model of the human B1AR was built using Modeller v8 [33] after alignment of the human and turkey sequences.

### Collective Variables (CVs)

The collective variables used in these simulations were the same as previously described in our earlier publications [19], [20], and are illustrated here in insets of Fig. 1 for each simulated dimeric arrangement of protomers *a* and *b*. Briefly, these CVs correspond to (i) the distance, *r*, between the centers of mass C*_a_* and C*_b_* of the TM regions of protomers *a* and *b*; (ii) the rotational angle, *θ_a_*, defined as the arccosine of the inner product of the normalized vectors connecting the projections onto the plane of the membrane of the centers of mass of the specific TM(s) at the interface (i.e., TM4/3 and TM1/H8) and of the two TM bundles (C*_a_* and C*_b_*), and (iii) the equivalent rotational angle, *θ_b_*, for the second protomer. To aid simulation convergence, we restricted the exploration of *θ_a_* and *θ_b_*, using steep repulsive potentials, the details of which are in the SI, Table S2.

### Biased MD Simulations

All simulations were performed using GROMACS version 4.0.5 [34] enhanced with the PLUMED plugin [35], and the system components were represented using the MARTINI forcefield [36], [37], [38] (using the parameters from version 2.1 for the protein beads, and version 2.0 for POPC and cholesterol), as described in our previous publications [19], [20]. We focused on two dimeric interfaces that have received experimental validation under physiological conditions according to recent publications on GPCRs, specifically those involving TM4 or TM1. The resulting dimeric configurations are illustrated by cartoons in the insets of Fig. 1, and correspond to TM4/3 and TM1/H8 interfaces. Thus, initial configurations were built for homodimers of B1AR or B2AR, as described in our previous publications [19], [20]. Subsequently, the dimers were converted to CG representation and embedded in a pre-equilibrated CG POPC/10% cholesterol membrane; the system was then solvated and counterions were added to neutralize the charge and to generate a physiological salt concentration of 0.1 M. An elastic network was used to restrain the protein system according to the strategy described in our previous publication [19]. Briefly, standard secondary structure constraints were introduced as per the MARTINI prescription; in addition, following a protocol put forward by Periole and colleagues [38], we introduced elastic potentials between beads within a cutoff of 9 Å to maintain the integrity of the protein tertiary structure. In a modification of the original implementation, the force constants of the elastic network were weaker on loops (250 kJ/mol) and stronger on the helical residues (1000 kJ/mol), with values chosen by matching the Cα fluctuations to those of a 50 ns, all-atom simulation of the same system [19]. The mean and standard deviation of the RMSD of the TM regions, and the whole receptor, calculated over all the simulations and reported in Table S3 for each system, demonstrate that the proteins maintained reasonably native conformations in the TM regions.

Metadynamics simulations [39] were carried out to generate the starting configurations for the umbrella sampling [40] simulations. During these metadynamics simulations, Gaussian bias was only applied to the CV describing the distance between protomers (*r* up to values representative of a protomeric system, see Table S2), and the CVs describing the relative rotation of the protomers (*θ_a_* and *θ_b_*) were restrained to ensure the starting structures all corresponded to interactions at the interface of interest only. Thus, we limited the sampling of the two rotational angles, *θ_a_* and *θ_b_*, to a predefined interval using upper and lower steep repulsive restraining potentials. Specifically, the upper and lower limits of this interval were set equidistant either side of the starting values from the initial dimeric configurations (see Table S1 for the range of *θ_a_* and *θ_b_*).

Approximately 40 umbrella sampling windows, were prepared for each of the dimeric systems with a force constant of 2400 kcal/(mol⋅nm^2^), (see Table S2 for range of separation, *r*), and extra windows were included at values of *r* where the reweighted distribution of the distances was found to be insufficiently sampled. We combined umbrella sampling [40] with well-tempered metadynamics [39] simulations to ensure thorough exploration of both the distance and angle space available to the system, for each of the windows. Thus, in addition to the constant external harmonic bias of the umbrella sampling algorithm applied to CV_1_ (i.e., *r*), a time-dependent sum of Gaussian biases in the well-tempered metadynamics algorithm was applied to the angle CVs (i.e., *θ_a_* and *θ_b_*).

In contrast to standard metadynamics, the bias potential in well-tempered metadynamics does not fully compensate the free energy surface, but rather depends on the underlying bias, decreasing to zero when a given energy threshold is reached [39]. Thus, not only does the computational effort remain focused on the physically relevant regions of the conformational space in these simulations, but also the convergence of the algorithm to a correct free energy profile can be proven rigorously. To ensure proper sampling, we checked that the chosen CVs could diffuse across one Gaussian size within the deposition time, so that local instantaneous equilibrium of the CVs would be satisfied. An improper choice of the bias update rate and Gaussian size would have resulted in trajectories failing to show multiple transitions across different minima and dependence on the initial starting configuration of the systems. None of the above was observed in our simulations, which showed instead increasingly fast diffusion of the CV dynamics due to the flattening of the underlying free energy surface, suggesting a proper sampling of the phase space of the systems. In all cases, the initial height of the biasing Gaussians was set to 0.12 kcal/mol, with a deposition stride of 10 ps, σ_M_ = 0.035 radians, and a bias factor of 15. Each window simulation was run for at least 1 µs (and up to 2 µs in regions of *r* thought to require additional sampling), resulting in a cumulative simulation time of ∼40 µs for each system, and a total of approximately 160 µs for the two receptor systems.

Finally, using a model potential, we provide (in the SI section) validation that the accuracy of the method combining umbrella sampling and metadynamics is comparable to those of standard multidimensional umbrella sampling or metadynamics (see both corresponding SI text and Fig. S5).

### Reweighting to Remove the Metadynamics Bias

The well-tempered metadynamics bias acting on the angle CVs distorts the probability distribution of the distance CV, thus requiring reweighting before equilibrium Boltzmann distributions could be reconstructed with WHAM. To recover the unbiased probability distribution of the distance CV from well-tempered metadynamics, we used the reweighting algorithm originally derived in [35] and direct the reader to the SI section for a description of this algorithm, as well as for the parameters used and additional technical details.

### Reconstruction of the Free Energy Surfaces

After recovering the unbiased probability distribution of the distance CV from well-tempered metadynamics, we used the well-documented WHAM technique [41], [42] to reconstruct, for each simulated system, the free energy surfaces as a function of the separation, *r*, between protomers (Fig. 1); the technical details are provided in the SI section. An error analysis of the reconstructed free energies was carried out combining recently proposed methods for the error estimation in umbrella sampling [43] and well-tempered metadynamics [44], [45] simulations. Equations used to estimate these errors are reported in the SI section. For each of the dimers at the primary minima (in Fig. 1), we have also reconstructed the FES as a function of the angles *θ_a_* and *θ_b_*. To reweight the angle distribution at a fixed protomer separation *r*, i.e., to remove the umbrella bias and obtain unbiased free energies as a function of the angles, we used the same algorithm as above [35], but we accounted for the umbrella potential as an external potential. Fig. S3 shows the FES as a function of (*θ_a_*,*θ_b_*) for the homodimers of the receptors at the two different interfaces. The minima marked Θ1, Θ1' and Θ2 are the principal minima from which representative minimum structures were extracted.

### Converting CG Structures Back to Atomistic Representations

Upon derivation of the FES as a function of *r* and the angles (*θ_a_*,*θ_b_*), for each dimeric system, we extracted a representative frame from the trajectory that corresponded to the minima therein. We then used Pulchra [46] to convert each of the CG models to an atomistic representation. These representations were solvated in an atomistic POPC/10% cholesterol membrane, energy minimized and simulated with harmonic restraints of decreasing strength applied to Cα atoms for a few picoseconds. Where necessary, we used an adiabatic biased MD simulation (of ∼5 ns) to improve the integrity of the helical structure of the re-converted receptors, by steering the Cα of the transmembrane regions toward the original atomistic structure of the B1AR or B2AR (i.e., that prior to coarse-graining), before 1 ns of unrestrained, all-atom simulation, during which the analyses to obtain the lists of contacts presented in Table S1 were conducted.

### Calculation of Thermodynamics Quantities

We can compare the strength of dimerization at each of the interfaces by the relative values of ΔG_X_°. In equation 1, we remind the reader of the formulation for ΔG_X_° in equation 5 of [19]. Specifically, the mole fraction standard free energy change can be expressed as:
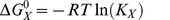
(1)where R is the universal gas constant, T is the temperature in Kelvin and K_X_ is the association equilibrium constant on the mole fraction concentration scale. Following our derivation in [19], K_X_ is approximately equal to:
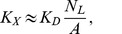
(2)where N_L_ is the number of lipids in the membrane of area A, and K_D_ is the dimerization constant expressed as in surface concentration units. For our membrane patch, *N_L_/A* was ≈1.65×10^6^ µm^−2^. Using the theory originally proposed by Roux and colleagues [47] and adapted by us to the case of GPCR dimers [19], the dimerization constant can be expressed as a function of the free energy *F(r)* of the system constrained to a predefined angular region Ω_0_, where *r* is the distance between the interacting protomers, Ω = (*θ_a_,θ_b_*) describes their relative orientation, and β = 1/*k*_B_T. This correction was necessary because the relative orientation between protomers had been constrained into a region Ω_0_. In such a case, by extending the integral up to the maximum distance *r*_D_ allowed for dimeric states, K_D_ is given by equation (3):
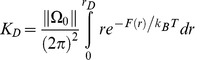
(3)Here, ||Ω_0_||, i.e., the product of the allowed ranges for *θ_a_* and *θ_b_* in radians (see SI and details in Table S2), is (maxθ-minθ-σ_M_)^2^≈0.51^2^≈0.26 at TM4/3 interfaces, and 0.46^2^ = 0.22 at TM1/H8 interfaces.

The above theory can be used to estimate the kinetics of dimerization by approximating the diffusion-limited association and dissociation rates, *k*_on_ and *k*_off_, at long timescales, following the Smoulchowski theory in two dimensions [48] (i.e., the membrane plane). This derivation is the same as that described in [19]. k_on_ is given by equation 4:
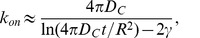
(4)where D_C_ is the sum of the diffusion constants of the two protomers, R is the sum of the protomer ratios, and γ is the Euler-Mascheroni constant and t refers to the experimental timescale of diffusion. Subsequently, k_off_ = k_on_/K_D_. An initial concentration of dimers of [D]_0_ evolves over time to give a concentration of:
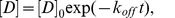
M(5)and thus the half-life of dimers can be estimated using:
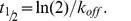
(6)


## Supporting Information

Figure S1 **Residency of lipid and cholesterol molecules around the interface.** Median (solid line) and 1^st^/3^rd^ quartiles (dotted lines) for the percentage of the trajectory spent within a minimum distance of 15 Å of the interface region for the PO4 headgroup of the POPC lipids (green) and the ROH moiety of the cholesterol molecules (black).
(PDF)

Figure S2 **Some long-resident cholesterol molecules visit similar positions to those observed in the crystal structures.** This figure shows the B2AR crystal structure superimposed on the CG structure of the dimer in the first frame of the 1 µs trajectory (after fitting to protomer 1 shown in dark gray) at the TM1/H8 interface, at *r* = 3.70 nm (i.e. the dimeric minimum). We observed that some of the small number of long-resident cholesterols near to the TM1/H8 interface (illustrated here by the ROH bead only, colored by residue, one bead per frame, over the whole trajectory), congregated in similar positions to the cholesterol molecules found in the crystal structures of B2AR (PDB ID: 2RH1,yellow sticks) (e.g. blue, purple beads), although not exclusively (e.g. red and green beads). Although much of the trajectory is spent close to the interface, there is some diffusion around the interface region (green, blue and purple beads). Some shorter resident cholesterol molecules also visit the region, but diffuse out quickly, indicated by the small number of beads (orange).
(PDF)

Figure S3 **Free energy surface as a function of** ***θ_a_*** **and** ***θ_b_*** **for the simulated adrenergic receptor homodimers.** The FES is shown for the minima, in (*r*) from Fig. 1 in the main text. Θ1, Θ1' and Θ2 indicate the principal minima in each surface, from which the representative minimum structures were extracted. The global minimum in each surface is assigned a value of zero energy and the remainder of the surface is colored according to its energy relative to the global minimum, in kcal/mol.
(PDF)

Figure S4 **Inter-protomer contacts highlighted for each interface.** Panels show the inter-protomer contacts (as listed in Table S1) for symmetrical contacting residues in the TM regions of opposing protomers where the interface is composed of (A) TM4/3 and (B) TM1/H8. Only one protomer of the dimer is given. B1AR is shown in red and B2AR is shown in blue. The inset panels in panel A show the TM4 region for the minima Θ2 (B1AR, salmon pink; B2AR, light blue), whilst the full protomer is shown for minima Θ1 for both receptor types. Contacting residues are labeled according to the Ballesteros-Weinstein numbering scheme and are colored according to the average separation (*x*) of their Cβ atoms, during 1 ns of unrestrained atomistic simulation. *x*<7 Å is shown in white spheres, 7 Å≤*x*<8 Å is shown as light grey spheres, 8 Å≤*x*<9 Å is shown as mid-grey spheres, 9 Å≤*x*<10 Å is shown in dark grey spheres, and 10 Å≤*x*<11 Å is black.
(PDF)

Figure S5 **Plots depicting reconstruction of the free energy surface of a known potential.** From A) 2-dimensional umbrella sampling, B) 2-dimensional metadynamics, and C) umbrella sampling combined with metadynamics. The exact numerical free energy is reported as a blue line in each panel.
(PDF)

Table S1 **Average contact distance between symmetric residues on opposing protomers during 1 ns of unrestrained simulation.** Inter-protomer residue contacts are defined for an example structure for each system (B1AR, B2AR at TM1/8 and TM4/3) extracted from the (*r*, *θ_a_*, *θ_b_*) minima from the coarse-grained simulations, reconverted to an atomistic representation, embedded into an atomistic and explicitly solvated POPC/10% cholesterol bilayer, and equilibrated and then simulated for 1 ns. The contacts are symmetric, defined between the Cβ of residues in the transmembrane regions only, on the opposing protomers. In addition to the sequence position, the Ballesteros-Weinstein numbering is also given. For the TM1/H8 interface, the closest interactions in H8 occur for residue R333 in the B2AR at 13 Å and in the B1AR, for the equivalent R384.
(PDF)

Table S2 **Range of collective variables used for each simulated receptor.** The range of *θ* is symmetric for both *θ_a_* and *θ_b_*.
(PDF)

Table S3 **The mean and standard deviation of the RMSD (in nm) of the TM regions, and the whole receptor, calculated over all the simulations.**
(PDF)

Text S1 **Supplementary file.** Additional methodological details.
(PDF)

## References

[1] Pin JP, Kniazeff J, Liu J, Binet V, Goudet C, et al (2005) Allosteric functioning of dimeric class C G-protein-coupled receptors. FEBS J 272: 2947–2955. 15955055 10.1111/j.1742-4658.2005.04728.x

[2] Milligan G (2008) A day in the life of a G protein-coupled receptor: the contribution to function of G protein-coupled receptor dimerization. Br J Pharmacol 153 Suppl 1: S216–229. 17965750 10.1038/sj.bjp.0707490 PMC2268067

[3] Chabre M, le Maire M (2005) Monomeric G-protein-coupled receptor as a functional unit. Biochemistry 44: 9395–9403. 15996094 10.1021/bi050720o

[4] James JR, Oliveira MI, Carmo AM, Iaboni A, Davis SJ (2006) A rigorous experimental framework for detecting protein oligomerization using bioluminescence resonance energy transfer. Nat Methods 3: 1001–1006. 17086179 10.1038/nmeth978

[5] Salahpour A, Masri B (2007) Experimental challenge to a ‘rigorous’ BRET analysis of GPCR oligomerization. Nat Methods 4: 599–600. 17664941 10.1038/nmeth0807-599

[6] Gurevich VV, Gurevich EV (2008) GPCR monomers and oligomers: it takes all kinds. Trends Neurosci 31: 74–81. 18199492 10.1016/j.tins.2007.11.007 PMC2366802

[7] Bayburt TH, Leitz AJ, Xie G, Oprian DD, Sligar SG (2007) Transducin activation by nanoscale lipid bilayers containing one and two rhodopsins. J Biol Chem 282: 14875–14881. 17395586 10.1074/jbc.M701433200

[8] Meyer BH, Segura JM, Martinez KL, Hovius R, George N, et al (2006) FRET imaging reveals that functional neurokinin-1 receptors are monomeric and reside in membrane microdomains of live cells. Proc Natl Acad Sci U S A 103: 2138–2143. 16461466 10.1073/pnas.0507686103 PMC1413699

[9] Whorton MR, Jastrzebska B, Park PS, Fotiadis D, Engel A, et al (2008) Efficient coupling of transducin to monomeric rhodopsin in a phospholipid bilayer. J Biol Chem 283: 4387–4394. 18033822 10.1074/jbc.M703346200 PMC2651572

[10] Hern JA, Baig AH, Mashanov GI, Birdsall B, Corrie JET, et al (2010) Formation and dissociation of M-1 muscarinic receptor dimers seen by total internal reflection fluorescence imaging of single molecules. Proc Natl Acad Sci U S A 107: 2693–2698. 20133736 10.1073/pnas.0907915107 PMC2823895

[11] Kasai RS, Suzuki KG, Prossnitz ER, Koyama-Honda I, Nakada C, et al (2011) Full characterization of GPCR monomer-dimer dynamic equilibrium by single molecule imaging. J Cell Biol 192: 463–480. 21300851 10.1083/jcb.201009128 PMC3101103

[12] Dorsch S, Klotz KN, Engelhardt S, Lohse MJ, Bunemann M (2009) Analysis of receptor oligomerization by FRAP microscopy. Nat Methods 6: 225–230. 19234451 10.1038/nmeth.1304

[13] Fung JJ, Deupi X, Pardo L, Yao XJ, Velez-Ruiz GA, et al (2009) Ligand-regulated oligomerization of beta(2)-adrenoceptors in a model lipid bilayer. EMBO J 28: 3315–3328. 19763081 10.1038/emboj.2009.267 PMC2748299

[14] Guo W, Urizar E, Kralikova M, Mobarec JC, Shi L, et al (2008) Dopamine D2 receptors form higher order oligomers at physiological expression levels. EMBO J 27: 2293–2304. 18668123 10.1038/emboj.2008.153 PMC2529367

[15] Knepp AM, Periole X, Marrink SJ, Sakmar TP, Huber T (2012) Rhodopsin forms a dimer with cytoplasmic helix 8 contacts in native membranes. Biochemistry 51: 1819–1821. 22352709 10.1021/bi3001598 PMC3332060

[16] Filizola M (2010) Increasingly accurate dynamic molecular models of G-protein coupled receptor oligomers: Panacea or Pandora's box for novel drug discovery? Life Sci 86: 590–597. 19465029 10.1016/j.lfs.2009.05.004 PMC2848910

[17] Wu B, Chien EY, Mol CD, Fenalti G, Liu W, et al (2010) Structures of the CXCR4 chemokine GPCR with small-molecule and cyclic peptide antagonists. Science 330: 1066–1071. 20929726 10.1126/science.1194396 PMC3074590

[18] Manglik A, Kruse AC, Kobilka TS, Thian FS, Mathiesen JM, et al (2012) Crystal structure of the mu-opioid receptor bound to a morphinan antagonist. Nature 485: 321–326. 22437502 10.1038/nature10954 PMC3523197

[19] Provasi D, Johnston JM, Filizola M (2010) Lessons from Free Energy Simulations of delta-Opioid Receptor Homodimers Involving the Fourth Transmembrane Helix. Biochemistry 49: 6771–6776. 20617813 10.1021/bi100686t PMC2914489

[20] Johnston JM, Aburi M, Provasi D, Bortolato A, Urizar E, et al (2011) Making Structural Sense of Dimerization Interfaces of Delta Opioid Receptor Homodimers. Biochemistry 50: 1682–1690. 21261298 10.1021/bi101474v PMC3050604

[21] Fonseca JM, Lambert NA (2009) Instability of a Class A G Protein-Coupled Receptor Oligomer Interface. Mol Pharm 75: 1296–1299. 10.1124/mol.108.053876 PMC2684881 19273553

[22] Chung KY, Rasmussen SG, Liu T, Li S, DeVree BT, et al (2011) Conformational changes in the G protein Gs induced by the beta2 adrenergic receptor. Nature 477: 611–615. 21956331 10.1038/nature10488 PMC3448949

[23] Wang H, Barreyro L, Provasi D, Djemil I, Torres-Arancivia C, et al (2011) Molecular determinants and thermodynamics of the amyloid precursor protein transmembrane domain implicated in Alzheimer's disease. J Mol Biol 408: 879–895. 21440556 10.1016/j.jmb.2011.03.028 PMC3082318

[24] de Jong DH, Periole X, Marrink SJ (2012) Dimerization of amino acid side chains: lessons from the comparison of different forcefields. J Chem Theory Comput 8: 1003–1014. 26593362 10.1021/ct200599d

[25] Sengupta D, Marrink SJ (2010) Lipid-mediated interactions tune the association of glycophorin A helix and its disruptive mutants in membranes. Phys Chem Chem Phys 12: 12987–12996. 20733990 10.1039/c0cp00101e

[26] Schafer LV, de Jong DH, Holt A, Rzepiela AJ, de Vries AH, et al (2011) Lipid packing drives the segregation of transmembrane helices into disordered lipid domains in model membranes. Proc Natl Acad Sci U S A 108: 1343–1348. 21205902 10.1073/pnas.1009362108 PMC3029762

[27] Periole X, Huber T, Marrink SJ, Sakmar TP (2007) G protein-coupled receptors self-assemble in dynamics simulations of model bilayers. J Am Chem Soc 129: 10126–10132. 17658882 10.1021/ja0706246

[28] Periole X, Knepp AM, Sakmar TP, Marrink SJ, Huber T (2012) Structural determinants of the supramolecular organization of G protein-coupled receptors in bilayers. J Am Chem Soc 134: 10959–10965. 22679925 10.1021/ja303286e PMC3406292

[29] Warne T, Serrano-Vega MJ, Baker JG, Moukhametzianov R, Edwards PC, et al (2008) Structure of a beta(1)-adrenergic G-protein-coupled receptor. Nature 454: 486–U482. 18594507 10.1038/nature07101 PMC2923055

[30] Cherezov V, Rosenbaum DM, Hanson MA, Rasmussen SGF, Thian FS, et al (2007) High-resolution crystal structure of an engineered human beta(2)-adrenergic G protein-coupled receptor. Science 318: 1258–1265. 17962520 10.1126/science.1150577 PMC2583103

[31] Wang C, Bradley P, Baker D (2007) Protein-protein docking with backbone flexibility. J Mol Biol 373: 503–519. 17825317 10.1016/j.jmb.2007.07.050

[32] Kandt C, Ash WL, Tieleman DP (2007) Setting up and running molecular dynamics simulations of membrane proteins. Methods 41: 475–488. 17367719 10.1016/j.ymeth.2006.08.006

[33] Fiser A, Sali A (2003) MODELLER: Generation and refinement of homology-based protein structure models. Methods Enzymol 374: 461–491. 14696385 10.1016/S0076-6879(03)74020-8

[34] Van der Spoel D, Lindahl E, Hess B, Groenhof G, Mark AE, et al (2005) GROMACS: Fast, flexible, and free. J Comput Chem 26: 1701–1718. 16211538 10.1002/jcc.20291

[35] Bonomi M, Branduardi D, Bussi G, Camilloni C, Provasi D, et al (2009) PLUMED: A portable plugin for free energy calculations with molecular dynamics. Comput Phys Commun 180: 1961–1972.

[36] Marrink SJ, Risselada HJ, Yefimov S, Tieleman DP, de Vries AH (2007) The MARTINI force field: Coarse grained model for biomolecular simulations. J Phys Chem B 111: 7812–7824. 17569554 10.1021/jp071097f

[37] Monticelli L, Kandasamy SK, Periole X, Larson RG, Tieleman DP, et al (2008) The MARTINI coarse-grained force field: Extension to proteins. J Chem Theory Comput 4: 819–834. 26621095 10.1021/ct700324x

[38] Periole X, Cavalli M, Marrink SJ, Ceruso MA (2009) Combining an Elastic Network With a Coarse-Grained Molecular Force Field: Structure, Dynamics, and Intermolecular Recognition. J Chem Theory Comput 5: 2531–2543. 26616630 10.1021/ct9002114

[39] Barducci A, Bussi G, Parrinello M (2008) Well-Tempered Metadynamics: A Smoothly Converging and Tunable Free Energy Method. Phys Rev Lett 100: 020603. 18232845 10.1103/PhysRevLett.100.020603

[40] Torrie GM, Valleau JP (1974) Monte-Carlo Free Energy Estimates Using Non-Boltzmann Sampling - Application to Subcritical Lennard-Jones Fluid. Chem Phys Lett 28: 578–581.

[41] Kumar S, Bouzida D, Swendsen RH, Kollman PA, Rosenberg JM (1992) The Weighted Histogram Analysis Method for Free Energy Calculations on Biomolecules. J Comput Chem 13: 1011–1021.

[42] Kumar S, Rosenberg JM, Bouzida D, Swendsen RH, Kollman PA (1995) Multidimensional Free Energy Calculations using the Weighted Histogram Analysis Method. J Comput Chem 16: 1339–1350.

[43] Zhu F, Hummer G (2012) Convergence and error estimation in free energy calculations using the weighted histogram analysis method. J Comput Chem 33: 453–465. 22109354 10.1002/jcc.21989 PMC3271861

[44] Berteotti A, Barducci A, Parrinello M (2011) Effect of urea on the beta-hairpin conformational ensemble and protein denaturation mechanism. J Am Chem Soc 133: 17200–17206. 21854002 10.1021/ja202849a

[45] Bonomi M, Barducci A, Parrinello M (2009) Reconstructing the equilibrium Boltzmann distribution from well-tempered metadynamics. J Comput Chem 30: 1615–1621. 19421997 10.1002/jcc.21305

[46] Rotkiewicz P, Skolnick J (2008) Fast procedure for reconstruction of full-atom protein models from reduced representations. J Comput Chem 29: 1460–1465. 18196502 10.1002/jcc.20906 PMC2692024

[47] Allen TW, Andersen OS, Roux B (2004) Energetics of ion conduction through the gramicidin channel. Proc Natl Acad Sci U S A 101: 117–122. 14691245 10.1073/pnas.2635314100 PMC314148

[48] Torney DC, McConnell HM (1983) Diffusion-Limited Reaction Rate Theory for Two-Dimensional Systems. Proc R Soc London 387: 147–170.

