# Supplementary figures and images for: Assessing the Relative Stability of Dimer Interfaces in G Protein-Coupled Receptors
Source: PLoS Comput Biol. 2012 Aug 16;8(8):e1002649. doi: 10.1371/journal.pcbi.1002649 (PMC3420924; doi:10.1371/journal.pcbi.1002649)

A. B1AR TM4/3

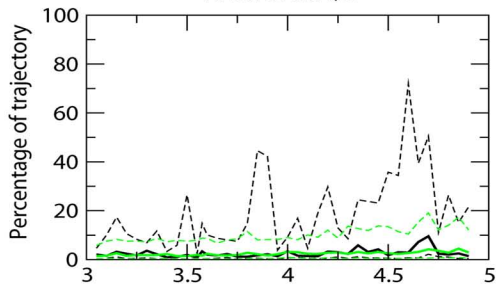

B. B2AR TM4/3

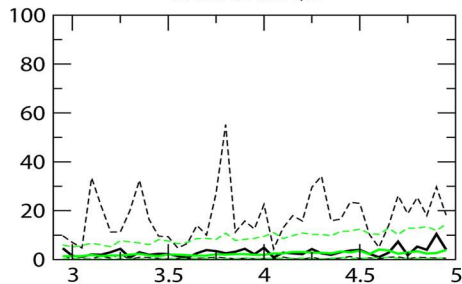

C. B1AR TM1/H8

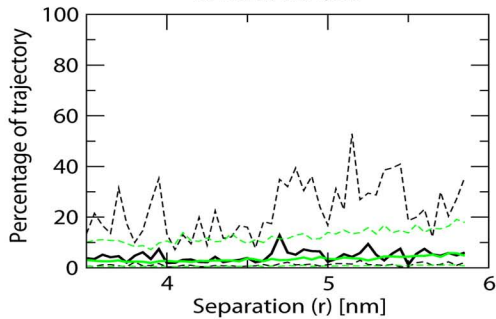

D. B2AR TM1/H8

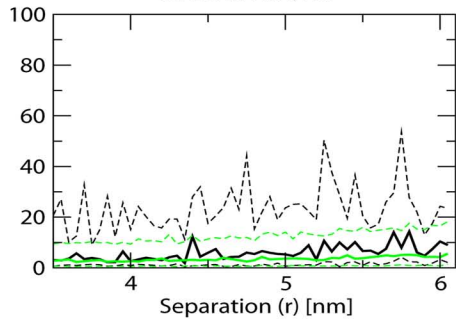

Supplement: Figure S1 — Residency of lipid and cholesterol molecules around the interface. Median (solid line) and 1st/3rd quartiles (dotted lines) for the percentage of the trajectory spent within a minimum distance of 15 Å of the interface region for the PO4 headgroup of the POPC lipids (green) and the ROH moiety of the cholesterol molecules (black). (PDF) [file pcbi.1002649.s001.pdf]

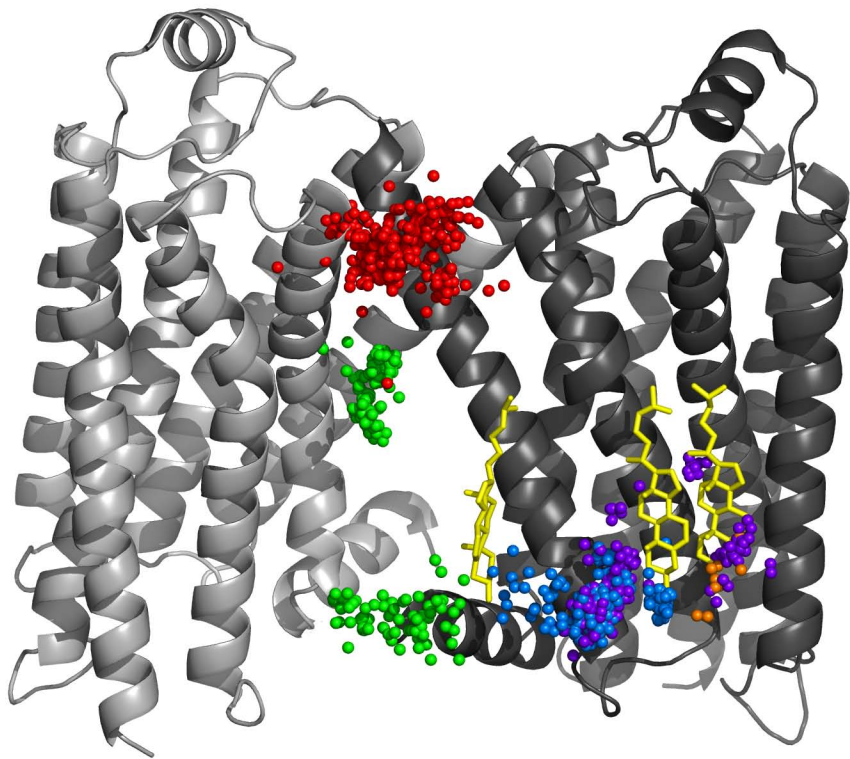

Supplement: Figure S2 — Some long-resident cholesterol molecules visit similar positions to those observed in the crystal structures. This figure shows the B2AR crystal structure superimposed on the CG structure of the dimer in the first frame of the 1 µs trajectory (after fitting to protomer 1 shown in dark gray) at the TM1/H8 interface, at r = 3.70 nm (i.e. the dimeric minimum). We observed that some of the small number of long-resident cholesterols near to the TM1/H8 interface (illustrated here by the ROH bead only, colored by residue, one bead per frame, over the whole trajectory), congregated in similar positions to the cholesterol molecules found in the crystal structures of B2AR (PDB ID: 2RH1,yellow sticks) (e.g. blue, purple beads), although not exclusively (e.g. red and green beads). Although much of the trajectory is spent close to the interface, there is some diffusion around the interface region (green, blue and purple beads). Some shorter resident cholesterol molecules also visit the region, but diffuse out quickly, indicated by the small number of beads (orange). (PDF) [file pcbi.1002649.s002.pdf]

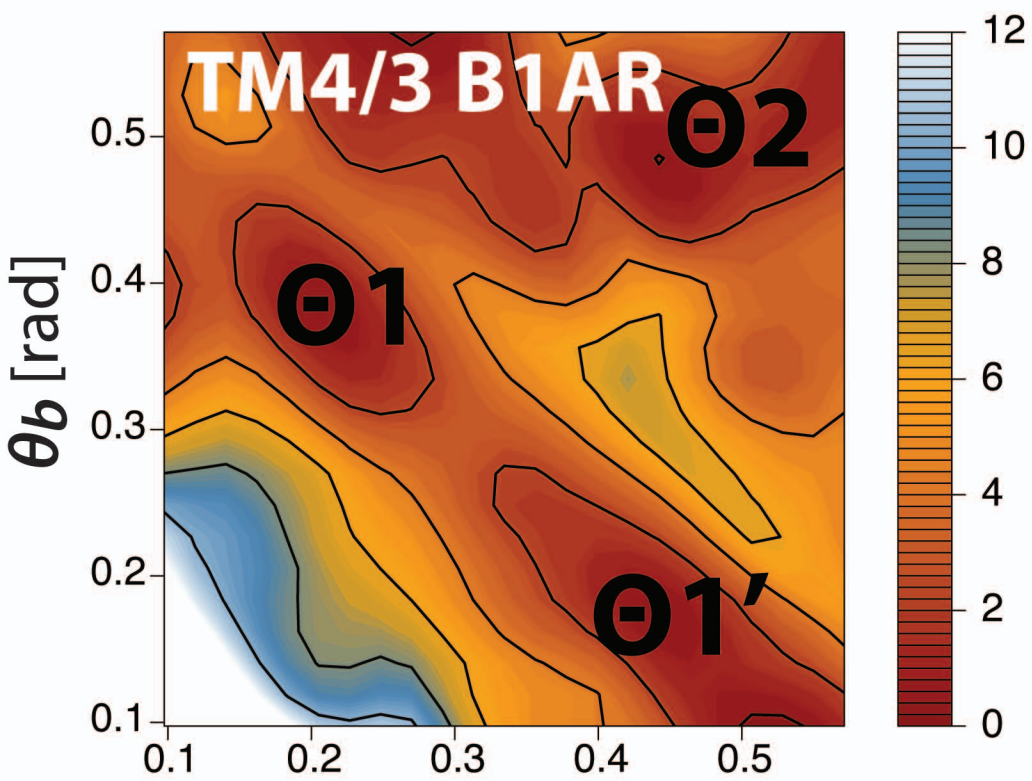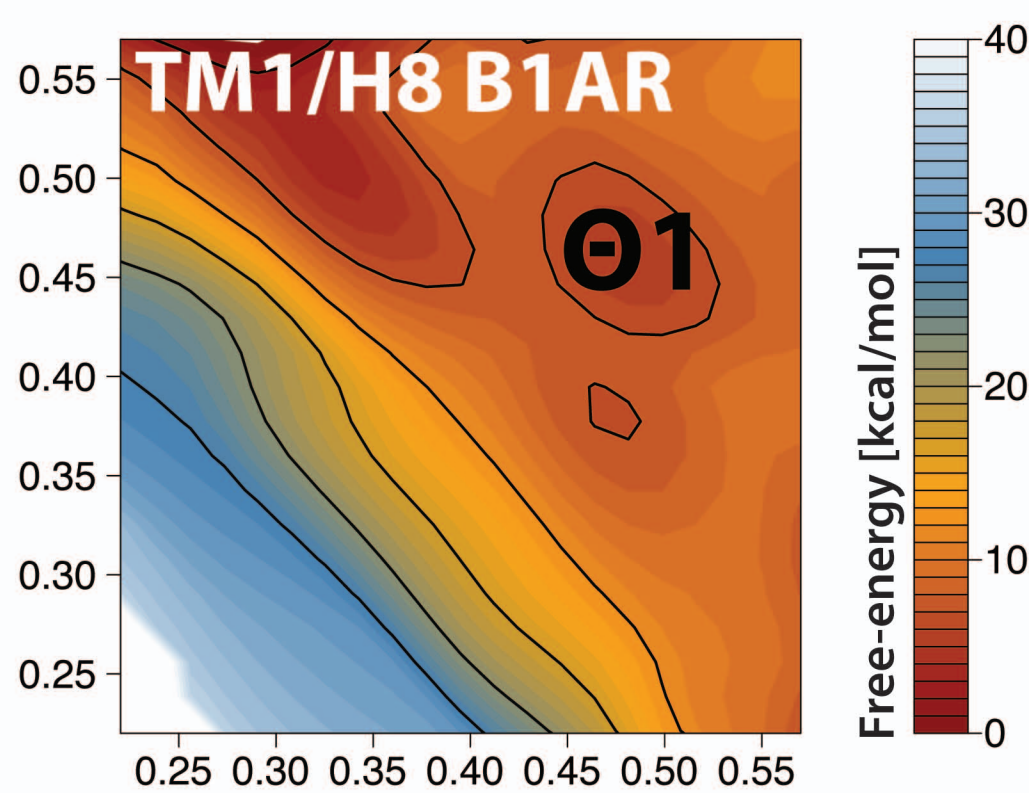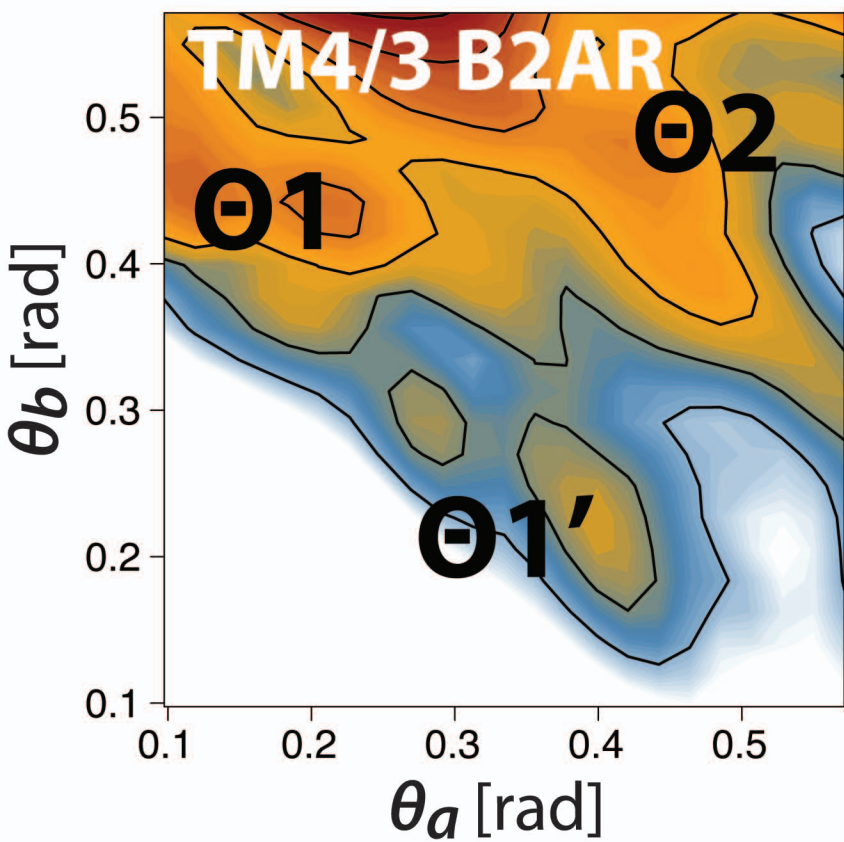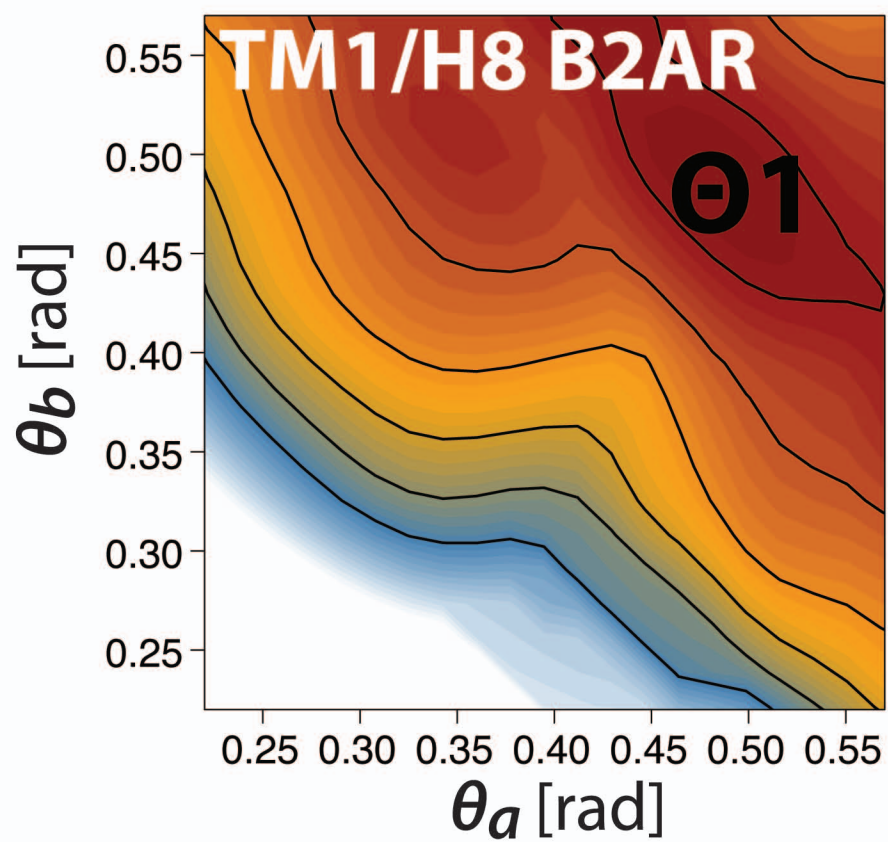

Supplement: Figure S3 — Free energy surface as a function of θa and θb for the simulated adrenergic receptor homodimers. The FES is shown for the minima, in (r) from Fig. 1 in the main text. Θ1, Θ1' and Θ2 indicate the principal minima in each surface, from which the representative minimum structures were extracted. The global minimum in each surface is assigned a value of zero energy and the remainder of the surface is colored according to its energy relative to the global minimum, in kcal/mol. (PDF) [file pcbi.1002649.s003.pdf]

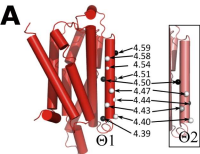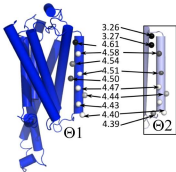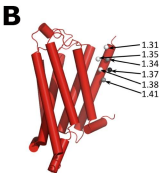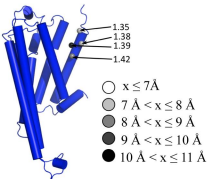

Supplement: Figure S4 — Inter-protomer contacts highlighted for each interface. Panels show the inter-protomer contacts (as listed in Table S1) for symmetrical contacting residues in the TM regions of opposing protomers where the interface is composed of (A) TM4/3 and (B) TM1/H8. Only one protomer of the dimer is given. B1AR is shown in red and B2AR is shown in blue. The inset panels in panel A show the TM4 region for the minima Θ2 (B1AR, salmon pink; B2AR, light blue), whilst the full protomer is shown for minima Θ1 for both receptor types. Contacting residues are labeled according to the Ballesteros-Weinstein numbering scheme and are colored according to the average separation (x) of their Cβ atoms, during 1 ns of unrestrained atomistic simulation. x<7 Å is shown in white spheres, 7 Å≤x<8 Å is shown as light grey spheres, 8 Å≤x<9 Å is shown as mid-grey spheres, 9 Å≤x<10 Å is shown in dark grey spheres, and 10 Å≤x<11 Å is black. (PDF) [file pcbi.1002649.s004.pdf]

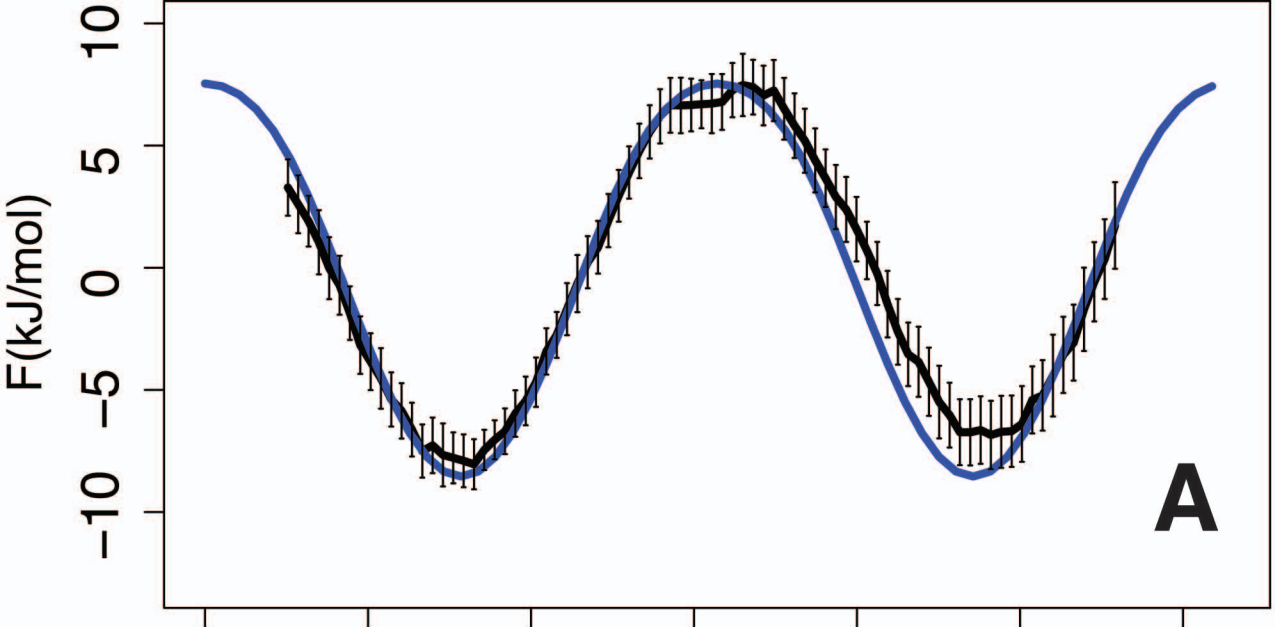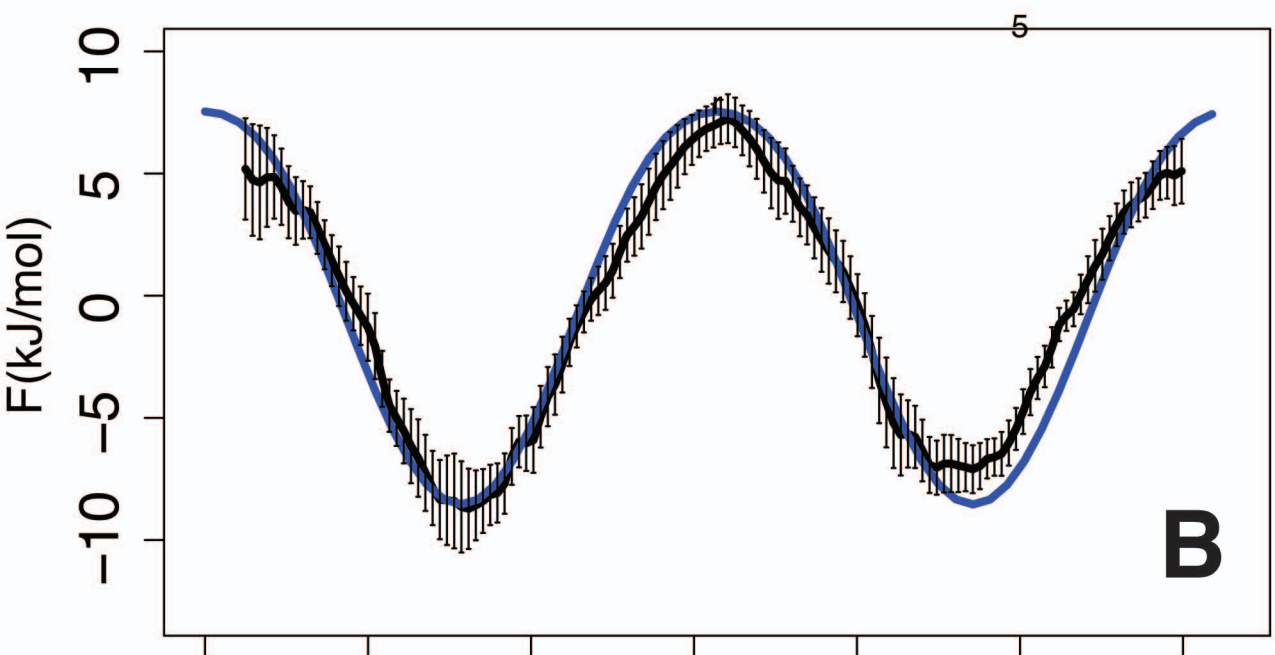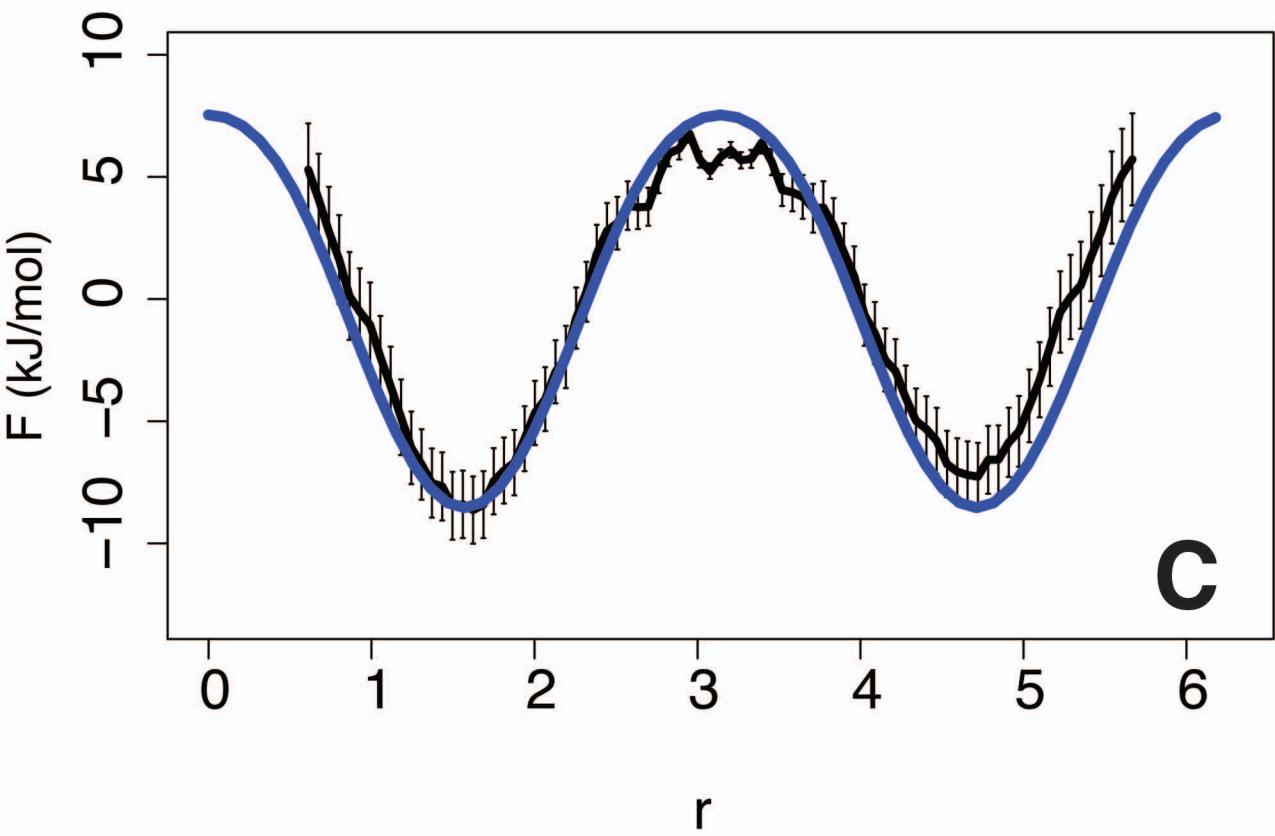

Supplement: Figure S5 — Plots depicting reconstruction of the free energy surface of a known potential. From A) 2-dimensional umbrella sampling, B) 2-dimensional metadynamics, and C) umbrella sampling combined with metadynamics. The exact numerical free energy is reported as a blue line in each panel. (PDF) [file pcbi.1002649.s005.pdf]
