# Supplementary material for: Assessing the Relative Stability of Dimer Interfaces in G Protein-Coupled Receptors
Source: PLoS Comput Biol. 2012 Aug 16;8(8):e1002649. doi: 10.1371/journal.pcbi.1002649 (PMC3420924; doi:10.1371/journal.pcbi.1002649)
Supplement: Table S1 — Average contact distance between symmetric residues on opposing protomers during 1 ns of unrestrained simulation. Inter-protomer residue contacts are defined for an example structure for each system (B1AR, B2AR at TM1/8 and TM4/3) extracted from the (r, θa, θb) minima from the coarse-grained simulations, reconverted to an atomistic representation, embedded into an atomistic and explicitly solvated POPC/10% cholesterol bilayer, and equilibrated and then simulated for 1 ns. The contacts are symmetric, defined between the Cβ of residues in the transmembrane regions only, on the opposing protomers. In addition to the sequence position, the Ballesteros-Weinstein numbering is also given. For the TM1/H8 interface, the closest interactions in H8 occur for residue R333 in the B2AR at 13 Å and in the B1AR, for the equivalent R384. (PDF) [file pcbi.1002649.s006.pdf]

Table S1. Average contact distance between symmetrical residues on opposing protomers during 1 ns of unrestrained simulation.

Distance (Å)	TM1/H8<br>B1AR	 B2AR	TM4/3<br>B1AR<br>**Θ1**	 <br>**Θ2**	 B2AR<br>**Θ1**	 <br>**Θ2**
≤ 7	W57$^{1.31}$<br>M61$^{1.35}$<br>L64$^{1.38}$	–	A173$^{4.40}$<br>R176$^{4.43}$<br>G177$^{4.44}$<br>C180$^{4.47}$<br>A187$^{4.54}$<br>F191$^{4.58}$	A173$^{4.40}$<br>C180$^{4.47}$	R151$^{4.43}$<br>N148$^{4.40}$<br>L155$^{4.47}$<br>F166$^{4.58}$<br>I159$^{4.51}$	N148$^{4.40}$<br>R151$^{4.43}$<br>L155$^{4.47}$
≤ 8	–	M36$^{1.35}$<br>V39$^{1.38}$	–	R176$^{4.43}$	V152$^{4.44}$	V152$^{4.44}$
≤ 9	G60$^{1.34}$<br>L67$^{1.41}$	–	A184$^{4.51}$	–	G162$^{4.54}$	K147$^{4.39}$
≤ 10	–	I43$^{1.42}$	R172$^{4.39}$<br>W183$^{4.50}$	G177$^{4.44}$	W158$^{4.50}$	I159$^{4.51}$<br>F166$^{4.58}$
≤ 11	L63$^{1.37}$	M40$^{1.39}$	L192$^{4.59}$	W183$^{4.50}$	I169$^{4.61}$	F108$^{3.27}$<br>E107$^{3.26}$

Inter-protomer residue contacts are defined for an example structure for each system (B1AR, B2AR at TM1/8 and TM4/3) extracted from the (r,θ_A,θ_B) minima from the coarse-grained simulations, reconverted to an atomistic representation, embedded into an atomistic and explicitly solvated POPC/10 % cholesterol bilayer, and equilibrated and then simulated for 1 ns. The contacts are symmetric, defined between the Cβ of residues in the transmembrane regions only, on the opposing protomers. In addition to the sequence position, the Ballesteros-Weinstein numbering is also given. For the TM1/H8 interface, the closest interactions in H8 occur for residue R333 in the B2AR at 13 Å and for the B1AR, the equivalent R384.
