# Supplementary material for: Assessing the Relative Stability of Dimer Interfaces in G Protein-Coupled Receptors
Source: PLoS Comput Biol. 2012 Aug 16;8(8):e1002649. doi: 10.1371/journal.pcbi.1002649 (PMC3420924; doi:10.1371/journal.pcbi.1002649)
Supplement: Table S2 — Range of collective variables used for each simulated receptor. The range of θ is symmetric for both θa and θb. (PDF) [file pcbi.1002649.s007.pdf]

Table S2. Range of collective variables used for each simulated receptor.

System	Interface	Range *r* (nm)	Range θ (rad)
B1AR	TM4/3	$3.00 \leq r \leq 4.90$	$0.05 \leq \theta \leq 0.60$
B2AR	TM4/3	$3.00 \leq r \leq 5.45$	$0.05 \leq \theta \leq 0.60$
B1AR	TM1/H8	$3.40 \leq r \leq 5.85$	$0.20 \leq \theta \leq 0.60$
B2AR	TM1/H8	$3.40 \leq r \leq 6.25$	$0.20 \leq \theta \leq 0.60$

The range of θ is symmetric for both θ_a and θ_b.
