# Supplementary material for: Assessing the Relative Stability of Dimer Interfaces in G Protein-Coupled Receptors
Source: PLoS Comput Biol. 2012 Aug 16;8(8):e1002649. doi: 10.1371/journal.pcbi.1002649 (PMC3420924; doi:10.1371/journal.pcbi.1002649)
Supplement: Table S3 — The mean and standard deviation of the RMSD (in nm) of the TM regions, and the whole receptor, calculated over all the simulations. (PDF) [file pcbi.1002649.s008.pdf]

Table S3. The mean and standard deviation of the RMSD (in nm) of the TM regions, and the whole receptor, calculated over all the simulations

	**TMs only**		**Whole protein**	
	**Protomer A**	**Protomer B**	**Protomer A**	**Protomer B**
B1AR, TM1/H8	0.09±0.009	0.08±0.010	0.12±0.010	0.10±0.010
B2AR, TM1/H8	0.10±0.017	0.09±0.011	0.15±0.022	0.13±0.018
B1AR, TM4/3	0.07±0.011	0.08±0.010	0.10±0.010	0.10±0.012
B2AR, TM4/3	0.10±0.014	0.09±0.012	0.15±0.025	0.16±0.025
