# Supplementary material for: Assessing the Relative Stability of Dimer Interfaces in G Protein-Coupled Receptors
Source: PLoS Comput Biol. 2012 Aug 16;8(8):e1002649. doi: 10.1371/journal.pcbi.1002649 (PMC3420924; doi:10.1371/journal.pcbi.1002649)
Supplement: Text S1 — Supplementary file. Additional methodological details. (PDF) [file pcbi.1002649.s009.pdf]

Supporting Information

Dynamic behavior of lipid and cholesterol at the dimerization interface

A GROMACS 'g_mindist' analysis was performed for each of the ~520 POPC molecules in the membrane patch, at each window, for each of the interfaces, for both the B1AR and B2AR systems. The percentage of each of the 1 μs-trajectories that each lipid headgroup (defined as the PO4 bead) spent within a minimum distance of 15 Å from the interface helices was calculated in each window. Between 250 and 350 POPC molecules spent a non-zero percentage of the trajectory within this cutoff distance. The median and $25^{th}/75^{th}$ percentiles of the residence times are plotted in Fig. S1 to indicate the mobility of these lipids at the interfaces. The same analysis was repeated for the hydroxyl moiety of each of the 52 cholesterol molecules in the membrane patch, for each window, for each interface, for both receptors. The same median and quartiles for the cholesterol molecules that spend a non-zero proportion of the trajectory within a 15 Å cutoff of the interface are also plotted in Fig. S1. For cholesterol, between 25 and 35 molecules spend some time within the cutoff. These data suggest that the residency of the cholesterol near the interface is generally longer than that for the POPC molecules. We observed that some of the small number of long-resident cholesterols (spending >85 % of their time within 15 Å of the interface) congregated in similar positions to the cholesterol molecules found in the crystal structures of B2AR (PDB IDs: 2RH1, 3D4S, and 3PDS), although not exclusively. Although much of the trajectory is spent close to the interface, there is some diffusion around the interface region. Fig. S2 shows the TM1/H8 interface for B2AR at r=3.70 nm as an example.

Reweighting algorithm

Herein, we provide a brief description of the reweighting algorithm that we used to recover the unbiased probability distribution (*P(r)*) of the distance collective variable (CV) *r*, centered at r_i, from the time-dependent sum of Gaussian biases *V(s,t)* deposited by well-tempered metadynamics along the trajectory of the angle CVs (*s* for each of them). The algorithm is implemented in a computer code using Plumed routines [1], and it is available for download. We refer the reader to [2] for the original mathematical derivation of this algorithm along with a validation of the method, estimates of the error in the reconstructed distributions, and a proof that this error decays with time as $t^{-1/2}$, ruling out systematic biases. Broadly, the algorithm consists of an adaptation of the relation typically used to unbias umbrella sampling simulations to time-dependent biases of the type introduced by well-tempered metadynamics.

The *biased* probability distribution at time *t* for a system in instantaneous equilibrium under the action of both an internal potential *W(x)* and the bias *V (s(x), t)* can be expressed as:

$$P(x,t) = \frac{e^{-\beta(W(x)+V(s(x),t))}}{Z_{W+V}} \qquad (1)$$

where *x* are the coordinates of the atoms in the system, *V(s(x),t)* is the bias from well-tempered metadynamics, *W(x)* includes both the force-field contribution and the umbrella potential Vr_i, and the partition function is:

$$Z_{W+V} = \iint dx\, dS\, \delta\big(S - s(x)\big) e^{-\beta(W(x)+V(s(x),t))}$$

Letting $Z = \int dx\, e^{-\beta W(x)}$, the distribution of the collective variables S in the *unbiased* system is related to the free energy by:

$$P(S) = \frac{e^{-\beta F(S)}}{Z_S} = \frac{\int dx\, \delta(S - s(x)) e^{-\beta W(x)}}{Z}$$

where $Z_S = \int dS\ e^{-\beta F(S)}$, so that the partition function in equation (1) can be expressed in terms of S as:

$$Z_{W+V} = \frac{Z}{Z_S}\int dS\ e^{-\beta(F(S)+V(S,t))}$$

and, eventually, one can write:

$$P(x,t) = \frac{e^{-\beta W(x)}}{Z} \cdot \ e^{-\beta(V(s(x),t))} \cdot \frac{Z_S}{\int dS\ e^{-\beta(F(S)+V(S,t))}}$$

in which the last fraction does not depend on *x* and constitutes a time-dependent offset to the bias $e^{-\beta(c(t))}$, so that the biased probability distribution becomes:

$$P(x,t) = P_0(x) \ \cdot e^{-\beta(V(s(x),t)+c(t))} \qquad (2)$$

Equation 2 is the desired relation between the biased distribution at time *t* and the Boltzmann unbiased distribution. In anticipation of its practical use in the reweighting process, we use equation (2), and the relation $c' = -\langle V' \rangle$ (where the prime symbol denotes derivative with respect to time *t*) to derive the evolution of *P(x, t)* with time:

$$P'(x,t) = -\beta P(x,t)(V'(s(x),t) - \langle V'(s(x),t)\rangle$$

This last equation is used to update the estimate of the biased distributions, from which unbiased distributions can be obtained by inverting equation (2).

The reweighting was performed between fixed points, defined for each system by the maximum and minimum values reached by each of the CVs in each of the windows, obtaining the three-dimensional probability $P_{ri}(r,\theta_a,\theta_b)$ as a function of for both angles and distance simultaneously. To determine the optimal variables for reweighting, and, later, for reconstruction of the free energy surface using WHAM, i.e. bin size and window length, we performed a small-scale

parameter search. The probability distribution of *r* was then calculated by integrating over the angle range,

$$P_{r_i}(r) = \int_{\Omega'} d\theta_a \, d\theta_b P_{r_i}(r, \theta_a, \theta_b)$$

where Ω' indicates the angles excluding the region within ½ σ_M of the boundary, which was removed to overcome inaccuracy in the sampling of this region resulting from the steep repulsive potentials used to define the limits of the angle exploration.

Error analysis

Error analysis of the reconstructed free energies was carried out combining recently proposed methods for the error estimation in either umbrella sampling [3] or well-tempered metadynamics [2,4] simulations. First, we used the formulations described by Zhu and Hummer [3] that allow error estimation in umbrella sampling simulations based on the statistical error of the mean force in each individual window. Specifically, for a potential mean force $F(x)$ along a given collective variable, *r*, sampled with harmonic biasing potentials, $K(r\text{-}x_i)^2/2$, positioned at $x_i=x_0$, x_1, …, x_M, and a reference value of $F(x_M)=0$ in the umbrella window corresponding to a monomeric state, the variance in the free energy, given by the square of the cumulative statistical error, was shown by Zhu and Hummer [3] to be approximately:

$$var[F(r)] \approx K^2 \cdot \sum_{i=i_r}^{M} var(\bar{r}_i)(x_i - x_{i-1})^2 \qquad (3)$$

where $var(\bar{r}_i)$ is the squared standard error in the estimate of the mean of *r* in window *i*, and i_r is the index of the window closest to r. Although these variances are usually calculated from block averages of the collective variables [5], our distribution of *r* under the biasing umbrella is

obtained from the reweighting algorithm of well-tempered metadynamics since the standard error on the mean cannot be estimated by block-averaging. Thus, to obtain $var(\overline{r_i})$, we follow the approach described in [4], estimating the averages as weighted time averages:

$$\overline{r_i} \cong \sum_{\tau=1}^{T} W_\tau \langle r \rangle_{\tau,i} \quad (4)$$

where $\langle r \rangle_{\tau,i}$ is the average in the *i*-th window at time τ, calculated from the metadynamics-unbiased distribution

$$\langle r \rangle_{\tau,i} = \frac{\int r P_{r_i}(r,\tau) dr}{\int P_{r_i}(r,\tau) dr}$$

Given the demonstrated convergence properties of the reweighting algorithm [2], where the variance of the estimate decays linearly with time, we follow [4] and use $W_\tau \propto \tau$. Thus, equation (3) gives:

$$var(\overline{r_i}) \cong \frac{\sum_\tau W_\tau (\langle r \rangle_{\tau,i} - \overline{r_i})^2}{\sum_\tau W_\tau} \quad (5)$$

The combination of equations (5) and (4) gives our final expression for the error in the free energy.

Additional technical details concerning the calculation of free energy estimates

Histograms of the collective variables were stored using bins of 0.02 rad for each angle and bins of 0.00125 nm for the distance, and saving the resulting unbiased free energy every 25 ns. We used the choice (`-rewtype 1`) that estimates the marginal probability of the distance and a value of σ_G of 2.0 (`-gauss 2.0`). We checked that the results are robust to variations of the bin size and σ_G.

The resulting free energies as a function of the distance were converted to probability distributions and input to a version of the WHAM code from the Grossfield Lab [6], which we trivially modified to read histograms instead of raw trajectories (details of the modified lines are available upon request). The averages $\langle r \rangle_{\tau,i}$ were calculated for τ starting from τ = 50 ns and every 25 ns, and the error bars estimated using equation (3).

Root Mean Square Deviation (RMSD)

The RMSD (in nm) was calculated for the backbone (BAS) beads of the transmembrane regions and the whole protein, over the duration of the simulations in each window, for both protomers for each receptor. The mean and standard deviations are given in Table S3.

Strategy Validation

To validate the combined umbrella sampling/metadynamics strategy presented here against standard multi-dimensional umbrella sampling or metadynamics simulations, we apply it to a 2-dimensional (2D) model system described by a Langevin bead (at T=300 K, with mass 10^5 amu, and friction γ=0.5 10^5 amu/ps) moving in a known potential given by

$$U(r,s) = \varepsilon \cos^2(s+\pi/2) \sin^2(r) \quad (6)$$

where ε=20 kJ/mol, chosen to have two minima separated by a high barrier along both degrees of freedom. We integrated the Langevin equations with a timestep of 0.05 ps, and calculate the free energy as a function of *r*, as $F(r)=\int ds \exp(-F(r,s)/k_BT)$ integrating out the *s* degree of freedom. For comparison, the exact result, obtained by numerical integration of eq. (6), is reported as a solid blue line in all panels of Fig. S5. We wish to clarify that the efficiency of each particular method strongly depends on the system under study and the nature of the collective variables

involved. Thus, the purpose of this model exercise was not to demonstrate superior efficiency and/or accuracy of any particular method, but rather to show that our combined umbrella sampling/metadynamics approach provides results with accuracy comparable to the other tested methods.

A standard 2D umbrella sampling simulation was performed by evenly subdividing the range of *s* and *r* in 13 windows (169 windows for an overall simulation length of ~2.5 10^8 steps) and applying harmonic restraints with k=80 kJ/mol. Alan Grossfield's 2D WHAM was used to generate the complete free energy F(*r*,*s*) from the resulting probabilities, and the corresponding, integrated F(*r*) is presented as a black solid line in panel A of Fig. S5. To estimate the uncertainty in the reconstructed free energy, we applied the common bootstrap strategy by re-sampling 100 times and used the obtained variance as a measure of the squared standard error.

Standard 2D well-tempered metadynamics was performed for a total length of 10^8 steps (σ_r=σ_s=0.05; initial height: ω_0=1.0 kJ/mol; deposition length: 500 steps; and bias factor: 15). The corresponding free energy F(*r*,*s*) was reconstructed by summing the Gaussian contributions and used to obtain the 1-dimensional F(*r*) in panel B of Fig. S5 by numerical integration. Following the approach used in the error analysis of the main text, we used time weighted averages to estimate the error on the free energy.

Finally, the combined umbrella sampling/metadynamics method was applied by using 13 windows (total simulation length: 0.65×10^8 steps) along the *r* direction, and well-tempered metadynamics (σ_s=0.05; initial height: ω_0=1.0 kJ/mol; deposition length: 500 steps; and bias factor: 15). Errors in the reconstructed free energy were estimated using Equation (3). The results are reported in panel C of Fig. S5.

References

1. Bonomi M, Branduardi D, Bussi G, Camilloni C, Provasi D, et al. (2009) PLUMED: A portable plugin for free energy calculations with molecular dynamics. Comput Phys Commun 180: 1961-1972.

2. Bonomi M, Barducci A, Parrinello M (2009) Reconstructing the equilibrium Boltzmann distribution from well-tempered metadynamics. J Comput Chem 30: 1615-1621.

3. Zhu F, Hummer G (2012) Convergence and error estimation in free energy calculations using the weighted histogram analysis method. Journal of Computational Chemistry 33: 453-465.

4. Berteotti A, Barducci A, Parrinello M (2011) Effect of urea on the beta-hairpin conformational ensemble and protein denaturation mechanism. J Am Chem Soc 133: 17200-17206.

5. Flyvbjerg H, Petersen HG (1989) Error estimates on averages of correlated data J Chem Phys 91: 461-466.

6. Grossfield A "WHAM: the weighted histogram analysis method", version 2.0.6.
